# Patterns of MADS-box gene expression mark flower-type development in *Gerbera hybrida *(Asteraceae)

**DOI:** 10.1186/1471-2229-6-11

**Published:** 2006-06-09

**Authors:** Roosa AE Laitinen, Suvi Broholm, Victor A Albert, Teemu H Teeri, Paula Elomaa

**Affiliations:** 1 Department of Applied Biology, P.O.Box 27, 00014 University of Helsinki, Finland; 2 Natural History Museum, University of Oslo, P.O.Box 1172, Blindern, NO-0318 Oslo, Norway

## Abstract

**Background:**

The inflorescence of the cut-flower crop *Gerbera hybrida *(Asteraceae) consists of two principal flower types, ray and disc, which form a tightly packed head, or capitulum. Despite great interest in plant morphological evolution and the tractability of the gerbera system, very little is known regarding genetic mechanisms involved in flower type specification. Here, we provide comparative staging of ray and disc flower development and microarray screening for differentially expressed genes, accomplished via microdissection of hundreds of coordinately developing flower primordia.

**Results:**

Using a 9K gerbera cDNA microarray we identified a number of genes with putative specificity to individual flower types. Intrestingly, several of these encode homologs of MADS-box transcription factors otherwise known to regulate flower organ development. From these and previously obtained data, we hypothesize the functions and protein-protein interactions of several gerbera MADS-box factors.

**Conclusion:**

Our RNA expression results suggest that flower-type specific MADS protein complexes may play a central role in differential development of ray and disc flowers across the gerbera capitulum, and that some commonality is shared with known protein functions in floral organ determination. These findings support the intriguing conjecture that the gerbera flowering head is more than a mere floral analog at the level of gene regulation.

## Background

The inflorescences of *Gerbera hybrida *(Asteraceae) are composed of three different types of flowers (ray, trans, disc) that are tightly packed into a condensed, radially organized flower head (capitulum). The presence of morphologically different flower types within a single genotype makes gerbera a unique target for reproductive developmental studies, since the traditional eudicot model plants (*Arabidopsis, Antirrhinum *and *Petunia*) bear only single flower forms in their inflorescences. In gerbera, the first stages of development are morphologically similar in all flower types, with only the position of individual flower primordia in the developing capitulum conferring their developmental fate. However, later in development many morphological differences emerge. The most prominent difference between the flower types is in their sex expression. Gerbera is a gynomonoecious species bearing both female and hermaphroditic flowers in the same inflorescence. In the outer female ray and trans flowers, stamen development arrests to form rudimentary staminodes [1], whereas in the central disc flowers, anthers develop fully, produce pollen, and form a postgenitally fused structure that covers the carpel. Furthermore, the corollas (fused petals) of ray flowers are long and zygomorphic (bilaterally symmetrical), whereas those of disc flowers vary from short-petalled and less asymmetrical to actinomorphic (radially symmetrical) at the very center of the capitulum. Corolla size and color can vary continuosly in different gerbera varieties [2].

Admixtures of different types of flowers are common in species that bear capitula or otherwise dense inflorescences [3,4] and probably reflect a selective advantage of this type of organization. The gerbera capitulum apparently mimics a large single flower, with the brigthly colored, elongate ray flowers attracting pollinators to the center of the inflorescence where bisexual flowers are located. Flower head form within Asteraceae can vary from radiate to discoid, bearing at least ray and disc flowers, or cycles of disc flowers only [5]. The prevalence of different flower head forms varies among the different subfamilies and tribes of the Asteraceae [5,6]. The presence or absence of ray flowers seems to be under control of one or two major genes, but several modifier genes are also involved [reviewed in [6,7]]. Perhaps the best studied system in this aspect has been *Senecio*, where flower head type is principally controlled by the *RAY *locus, with the radiate phenotype dominant over discoid [8-10]. Gross phenotypic resemblance to the *centroradialis *(*cen*) mutant of *Antirrhinum*, which has a radially symmetrical terminal flower surrounded by zygomorphic axillary flowers in peripheral region around the inflorescence apex, has prompted research to test the hypothesis [11] that the *RAY *locus encodes a homolog of the floral symmetry gene *CYCLOIDEA *[12-14]. However, recent microarray comparisons of *Senecio *taxa differing primarily in flower morphology revealed only few genes – and not *CYCLOIDEA *– potentially involved in observed differences in floral development [15].

Progress in the genetics of plant reproductive development over the last two decades has shown that the most important genes specifying flower development encode transcription factors, many of them MADS domain proteins [16-18]. There are several examples in which homologous genes have evolved different functions in different species as the result of sub- or neofunctionalization [17]. Furthermore, relatively simple regulatory changes in transcription factors can be responsible for the evolution of new morphologies [e.g., [19-21]]. In this study we have morphologically characterized very early stages of ray and disc flower development in *Gerbera hybrida*. We compared gene expression profiles among three different stages of disc and ray flower development using a gerbera cDNA microarray that includes 9000 probes representing ESTs from different tissues and treatments [22]. Our goal was to gather an overall picture of the processes involved in early gerbera flower development by identifying genes that may function disparately in the differentiation pathways of ray versus disc flowers. Surprisingly, we found that MADS-box genes otherwise controlling floral organ determination were differentially expressed in the divergent flower types.

## Results

### Early stages of flower development in *Gerbera hybrida*

**Figure 1:**
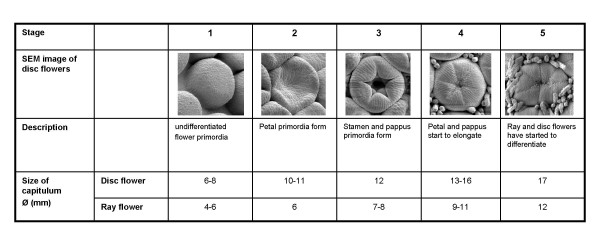
**Gerbera flower developmental stages from 1 to 5**. The different stages (1–5) of early flower development are shown with perfect disc flowers that are morphologically similar to ray flowers until stage 4. Stage 5 for ray flowers is shown in figure 2 A.

**Figure 2:**
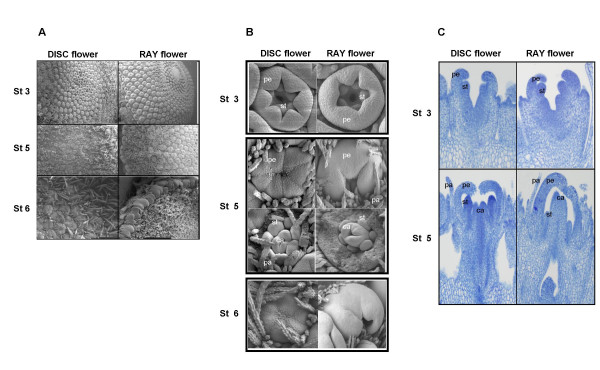
**Morphological analysis of the early stages of disc and ray flower development in gerbera**. **(A) **SEM images showing developing gerbera capitula when disc and ray flowers are at stages 3, 5 and 6, respectively. **(B) **SEM analysis comparing developmental stages 3, 5 and 6 in disc and ray flowers. Differences in stamen and in petal development between flower types start to emerge at stage 5. **(C) **Histological analysis of ray and disc flower primordia at stage 3 and 5. There is no difference in stamen development in ray and disc flowers at stage 3 while at stage 5 stamen development in ray flowers lag behind. Abbreviations: pa = pappus bristles, pe = petal, st = stamen, ca = carpel.

During early stages of reproductive development, individual flower primordia arise in an acropetal spiral from the inflorescence meristem. Undifferentiated ray flower primordia are the first to arise in inflorescences of less than 6 mm in diameter, whereas the first disc flower primordia are detected in inflorescences of approximately 6–8 mm in diameter (Figure 1). We divided early flower development into six different stages that were characterized using scanning electron microscopy (SEM) and histological staining (Figures 1 and 2). Figure 1 demonstrates the first five stages of perfect (fully bisexual) disc flower development. At stage 1, flower primordia are small, undifferentiated bumps, whereas at stage 2, ring-shaped petal primordia begin to form. At stage 3, pappus (whorl 1), petal (whorl 2) and stamen (whorl 3) primordia can be clearly distinguished. Until this point in development, both ray and disc flowers still appear morphologically similar and can only be identified based on their position in the inflorescence (Figure 2A). At stage 4, petals begin to elongate, covering the developing stamen and carpel primordia. Flower organ elongation continues during stages 5 and 6 (Figure 2B). As with SEM, histological analysis reveals no morphological differences between ray and disc flowers at stage 3 (Figure 2C). However, at stage 5, stamens lag behind and are shorter in the female ray flowers compared to the perfect disc flowers (Figures 2B and 2C). Furthermore, at stage 5, the ray flower petals have already fused together, and the shapes of the bilabiate ray flowers with their three highly extended lower lobes plus two minute upper lobes can be distinguished. Conversely, in disc flowers, the developing petals are separated from each other. At stage 6, the differences in petal elongation are even more pronounced. At this stage, pappus bristles are much longer in both flower types, nearly covering the developing primordia (Figures 2A and 2B). In earlier work, we defined the stages of capitulum development based on ray flower petal development [23]. Developmental stage 6 in ray flowers and disc flowers as described here corresponds to developmental stages 1 and 3 of the capitulum, respectively [23].

### Microarray analysis of gene expression during ray and disc flower development

cDNA microarray analysis comparing ray and disc flower primordia was performed separately for developmental stages 3, 5 and 6. The gerbera cDNA microarray contains approximately 9000 probes representing all distinct unigenes from the gerbera EST collection [22]. Most of the probes (ca. 80%) printed on the microarray represent transcripts from floral cDNA libraries and genes expressed during inflorescence development [22]. Microdissected material for RNA isolation was collected and pooled together from a single clone over a year's time from hundreds of flower primordia across tens of inflorescences. In circumstances such as with gerbera flower primordia sampling, where true biological replicates are not possible due to limited amounts of plant material, pooling is a means to minimize biological variation. For our experiments we also pooled several independent RNA isolations and amplified them prior to dye labelling. Each experiment included four technical replicates each with two dye-swaps. We have verified the present microarray results with real-time reverse transcriptase PCR for 12 random transcripts and for 8 gerbera MADS-box genes (See Additional files 1 and 2).

**Figure 3:**
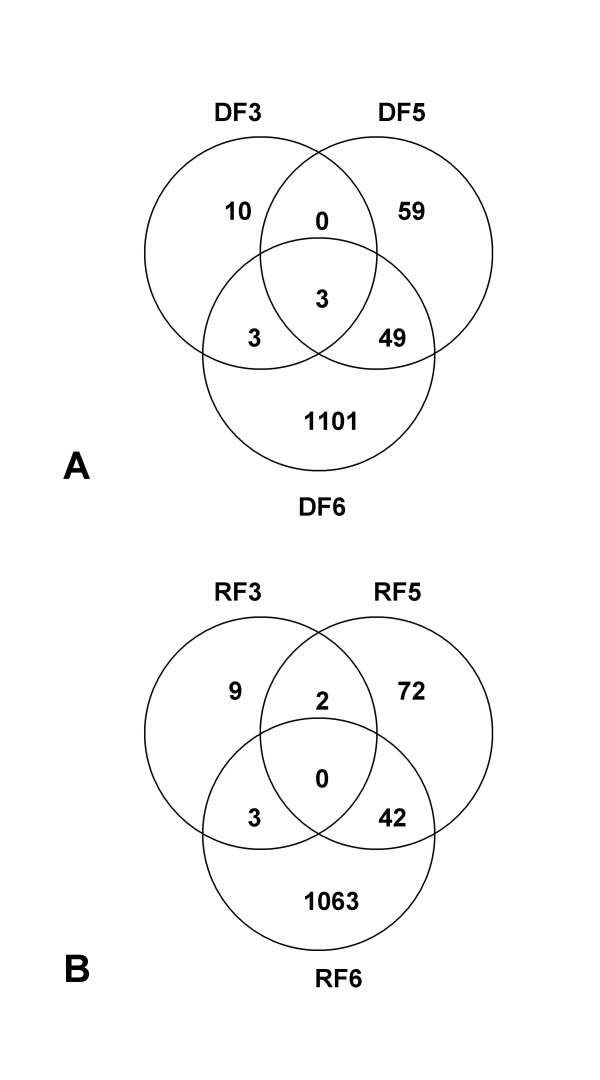
**Distribution of the differentially expressed genes**. Venn diagram shows distribution of genes which are significantly differentially expressed in different stages of individual flower types. Differential expression is based on one-sample t-test p-values. The number of differentially expressed genes increases with development and correlates with the morphological changes detected in flower types. Many of the genes show stage-specific expression. Genes expressed only at stage 3 and those common to stages 5 and 6 were analyzed more closely.

Following non-linear Lowess normalization of the microarray data, a statistical analysis (one-sample t-test) was performed to discover genes that show significant differences in their expression. The analysis was done separately for each developmental stage. To minimize the number of false positives a Benjamin-Hochberg false discovery rate (FDR) was used to control the Type I family-wise error. Transcripts with p-value < 0.05 were selected as statistically significant. With this analysis, the total numbers of differentially expressed genes were 29 at stage 3, 227 at stage 5, and 2264 at stage 6. Distributions of these genes within and between the flower types are visualized with the Venn-diagram shown in figure 3. Only few expressed genes were shared between stages, demonstrating the rapid transcriptional changes occurring during very early stages of gerbera flower development. The raw data for the experiments presented here have been lodged at the ArrayExpress under accession number E-MEXP-418.

### Genes differentially expressed during ray and disc flower development

At the earliest developmental stage included in the comparison (3), the disc and ray flowers were morphologically similar. This was reflected at the gene expression level, where few changes are detected. Only 27 transcripts showed significant differences in expression with p-value < 0.05 and fold change more than 1.2 (Table 1). From these, 15 showed greater expression in disc flowers and 12 in ray flowers. In disc flowers most of the genes were related to basic metabolic processes. One of the most abundant transcript upregulated in disc flowers is the gerbera homolog of Vegetative Storage Protein (VSP) gene. VSPs principally accumulate in vacuoles and have a putative role in nitrogen storage and in biotic and abiotic stress responses. In *Arabidopsis*, VSP mRNAs are highly abundant in flowers, where they may have distinct roles during reproductive development [24,25]. The gerbera VSP homolog also showed significantly greater expression in stage 6 disc flowers. In ray flowers at stage 3, most of the upregulated genes encoded unknown proteins. However, one gerbera MADS-box gene, *GRCD1*, showed greater upregulation. *GRCD1 *has previously been suggested to be required for defining stamen identity in gerbera [1].

The number of differentially expressed genes was found to increase with developmental time, in concordance with increasing morphological differences between the flower types. In disc flowers, the number of genes showing statistically significant changes in expression was 111 at stage 5 and 1156 at stage 6, and in ray flowers, 116 and 1108, respectively. Below, we limit further discussion to the group of genes showing flower-type specific upregulation during both stages 5 and 6 (Table 2). In disc flowers, 49 transcripts were upregulated in both stages 5 and 6 (Table 2). Twenty (41 %) of these gave a BLAST hit while the rest have unknown function.

One of the most significantly upregulated gene had a match to GDSL-lipase. These enzymes are highly abundant in *Arabidopsis *flowers and are postulated to have specific functions during anther morphogenesis [25,26]. GDSL-motif lipase genes are also highly expressed throughout tepal development in *Iris hollandica *[27]. Another upregulated gene encoded alpha-expansin, known to be involved in growth and development [28,29]. The most prominent group of genes, however, were those encoding lipid-transfer proteins, which form a large gene family in gerbera [22]. The gerbera C-class MADS-box gene GAGA1 was also more abundantly expressed in disc flowers in comparison to ray flowers at both stages 5 and 6.

42 genes were more strongly expressed in ray flowers than in disc flowers at both stages 5 and 6 (Table 2). Of these, 55 % (23), had unknown functions, and many others encoded ribosomal proteins. Most of the identified genes (36) were also upregulated during early petal development (in ray flowers) in our previous microarray analyses [22] reflecting differences in petal development that occur during early stages of flower-type differentiation.

*G0000600014B5 *(*GEG4*), a member of the GASA protein family (Kotilainen, unpublished results), was previously detected in elongating flower scapes in addition to being expressed during early petal development [22]. *G0000700014E10*, annotated as a polyphenol oxidase precursor, was more than 5-fold upregulated in ray flower primordia in comparison to disc flowers. Polyphenol oxidases (PPOs) are ubiquitous in higher plants and are the major cause for tissue browning due to oxidization of phenolic substrates. PPOs have been suggested to have a role in defense against insects and plant pathogens, but they may also be activated by mechanical stress. A PPO homolog encoded by aureusidin synthase has been shown to function in flower coloration [30].

### Transcription factor genes showing differential expression at stages 5 and 6

Since transcription factors widely regulate many aspects of development, we examined these genes more closely. In addition to the statistical criteria (p < 0.05) we used a fold change threshold of > 1.2 for differentially expressed transcripts. As described earlier, at stage 3, only the MADS-box gene *GRCD1 *was detected to have greater expression in ray flowers as compared with the corresponding stage in disc flowers. In both flower types, most of the differentially expressed transcription related genes were expressed during stages 5 and 6, and moreover, they showed stage-specific expression (Tables 3 and 4). Especially prominent was the upregulation of several homeotic MADS-box genes that are known to regulate flower development (discussed separately below).

In addition to MADS-box genes, we identified one gene differentially expressed in disc flowers at stage 5 encoding a putative RING zinc finger protein. RING and variant-RING domain proteins are widespread in plants, and as parts of multiprotein complexes they are involved in diverse cellular functions, e.g., ubiquitination pathways [31,32]. Two gerbera homeobox HD-ZIP factor genes upregulated in disc flowers shared high sequence similarity with a xylem-specific *Zinnia elegans *gene [33] as well as with *Arabidopsis REVOLUTA *(*REV*) and *CORONA *(*CNA*) genes, both members of the HD-ZIP III gene family [34,35]. HD-ZIP III genes play numerous roles in development, including embryo patterning, vascular development, leaf development (organ polarity) as well as meristem initiation [35,36]. The gerbera EST database currently includes four clusters of genes that are homologous with Squamosa Promoter Binding Proteins (SBPs) and Squamosa Promoter Binding Like (SPL) proteins. Two of these were upregulated in disc flowers at stage 6. In *Arabidopsis*, *SPL3 *has been shown to be involved in floral transition [37,38] and *SPL8 *in pollen sac development [39] but functions for the other 16 *SPL *genes remain largely unknown. We also identified two genes encoding YABBY transcription factors (*G0000800003E02, G0000700008F1)*. Together with *KANADI *genes, the *YABBY *gene family promotes abaxial identity of organs [36]. In addition, several transcription factors related to regulation of transcription initiation, chromatin assembly, and mRNA processing were upregulated in disc flowers.

In ray flower primordia, 39 genes were differentially upregulated (Table 4). Many of the transcription factors therein belong to different classes of zinc-finger proteins [40]. BLAST searches indicated that *G0000100020B08 *and *G0000600011G06 *are homologous with putative flowering-time genes identified in maize (*INDETERMINATE*1, [41]) and *Arabidopsis *(*CONSTANS*-like *3*). The functional role for the GATA1 zinc-finger-like protein G0000500013B9 is not known as yet. We also identified a LIM domain protein homolog upregulated in ray flowers at stage 6. We have previously observed that pLIM2 [22] was more than 8-fold upregulated during late stages of stamen development. Nevertheless, multiple functions have been shown for LIM domain proteins in plants, including transcriptional regulation in the nucleus [42,43,40]. Finally, at stage 6, we detected upregulation of the bHLH factor encoded by *G0000600012D07*, previously isolated as *GMYC1 *and involved in anthocyanin regulation in gerbera [44].

### Several MADS-box genes show differential expression during ray and disc flower development

Several previously characterized gerbera MADS-box genes [[45,46] Figure 5] showed differential expression during the early stages of ray and disc flower differentiation (Tables 1, 2, 3 and 4). *GAGA1 *and *GAGA2 *are C-function genes known to be involved in regulation of stamen and carpel identity in gerbera [47]. *GDEF1 *shares high sequence similarity with *GDEF2*, another B-function gene in gerbera, but it groups phylogenetically with *TM6*-like genes of the B-class lineage [48]. Its function during gerbera flower development has not yet been established [45]. *GRCD2 *is required for carpel identity, but the gene also controls maintenance of flower meristem identity as well as inflorescence determinacy [49]. All of the aforementioned genes are upregulated in bisexual disc flowers in comparison to female ray flowers. PHEP7F1 encodes the SQUAMOSA-like gene *GSQUA1 *[47], and *G0000800002C9*, also upregulated in disc flowers, encodes a MADS-box gene homologous to *Chrysanthemum CDM8*, which belongs to the *FRUITFULL *clade [50].

In ray flowers, *GRCD1*, which was more expressed already at stage 3, was similarly upregulated at stage 6. In addition to *GRCD1*, another MADS-box gene, *G0000100021A03*, showed greater expression in ray flowers at stage 6. *G0000100021A3 *(including the 3' EST *G0000100002C11*) is a *TM3*-like MADS-box gene that showed late petal-specific expression in our previous microarray analyses [22]. *G0000700003A3 *(now named *GRCD5*), which was significantly more expressed in ray flowers, groups phylogenetically close to the previously identified gerbera gene *GRCD1 *as well as Arabidopsis *SEPALLATA3 *(data not shown). *GRCD5 *shares highest sequence similarity with the *CDM44 *MADS-box gene from chrysanthemum and *FBP2 *of petunia [50,51]. In fact, *GRCD5 *appears to be orthologous to *CDM44 *in phylogenetic analysis (data not shown). According to the same phylogenetic analysis (data not shown), *G0000200014A7 *(*GRCD3*), which is transcriptionally abundant in ray flowers, lies in the *AGL6 *clade [52], grouping close together with *AGL6*, *MDMADS11*, and *ZAG3 *and *ZAG5 *[cf. 1]. *G0000200001C06 *shows sequence similarity to the chrysanthemum *CDM51 *and gerbera *G0000500017F4 *MADS-box genes, the latter of which has a leaf specific expression pattern [22] similar to *Arabidopsis *AGAMOUS-like MADS-box protein *AGL12*. We observed similar expression patterns for the gerbera MADS-box genes using real time reverse transcriptase PCR (See Additional file 2).

### Co-expression of MADS-box genes during flower development in gerbera

**Figure 4:**
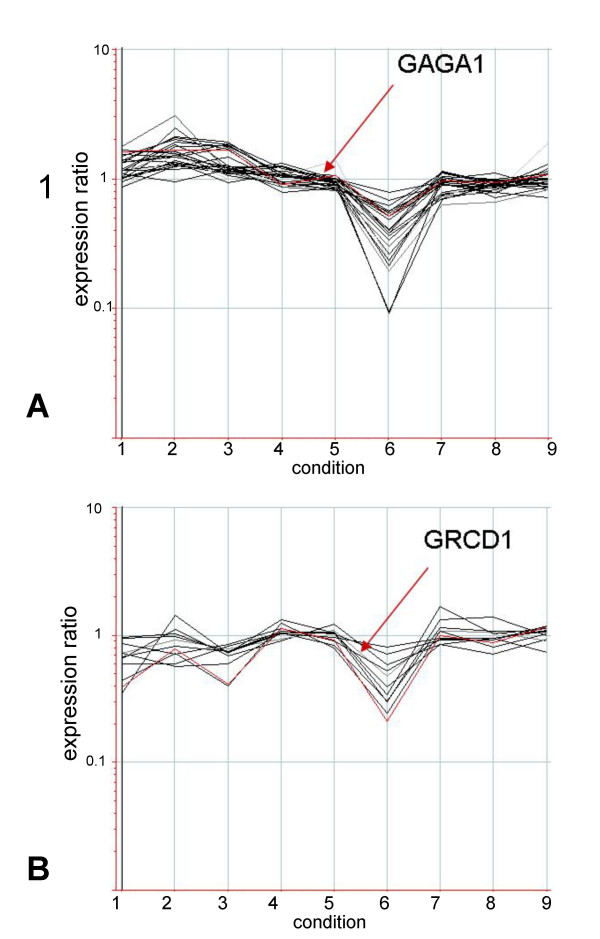
**Correlation with *GAGA1 *and *RCD1***. Graph showing genes classified as "transcription" that correlate more than 0.80 with **(A) ***GAGA1 *and **(B) ***GRCD1 *expression (indicated in red) in nine different experiments that were included in analysis. Conditions: 1 = DF/RF stage3, 2 = DF/RF stage 5, 3 = DF/RF stage 6, 4 = early petal, 5 = late petal, 6 = leaf/flower, 7 = pappus, 8 = flower scape and 9 = stamen).

MADS domain proteins form specific homo- and heterodimers and even higher order complexes to conduct their function [53,54]. Specific interactions among MADS domain proteins require that they are present in the same cells and tissues under the same developmental stages, and correspondingly, it has been shown that transcripts with overlapping expression patterns are preferred as protein interaction partners [55,56]. In order to strengthen our observations above and to reveal coordinated expression patterns for gerbera MADS-box genes, we combined our present observations with our previous analyses [22]. In an independent analysis we looked for genes that were differentially regulated along with *GAGA1 *and *GRCD1*. *GAGA1 *was chosen because it was differentially expressed in disc flowers at both stages 5 and 6, and *GRCD1 *due to its early upregulation in ray flowers at stage 3 (Tables 1 and 2). Previously [22], we used the gerbera cDNA microarray to identify inflorescence-specific genes in comparison to leaf tissue as well as genes specific for individual reproductive organs and stages (flower scape, pappus bristles, early and late petal development, and stamens). The samples represented relatively late stages of flower organ development in contrast to the early stages of flower primordium development analyzed here. Standard correlation was used to find transcription factors whose expression correlated (correlation coefficient >0.80) with *GAGA1 *or *GRCD1 *across all nine conditions (Figure 4). Table 5 summarizes the identified genes.

The first set of genes represents those that are co-expressed with the C-class gene *GAGA1*, which was the only MADS-box gene showing significant change in expression in both stages 5 and 6 (Table 2 and 3). Across all nine conditions included in the analysis, 27 genes showed an expression pattern similar to *GAGA1*. The highest correlation (0.96) was observed for *GRCD2*, the product of which was previously demonstrated to interact with *GAGA1 *using yeast two hybrid analysis [49]. Furthermore, the second gerbera C-function gene, *GAGA2*, as well as the B-function genes *GGLO1 *and *GDEF2*, were co-expressed with *GAGA1*. *GGLO1*, a B-function gene orthologous to Arabidopsis *PISTILLATA *required for petal and stamen identity [47]. also showed disc-flower specific expression at stages 3 and 5 in microarray (when multiple-testing correction was not applied). This result was verified using real time RT-PCR (Table S1b) and northern blotting (data not shown). The functionally unknown *TM6*-like gene *GDEF1*, the *SEPALLATA-*like gene *GRCD4*, and *GSQUA1 *were also included among the *GAGA1*-coexpressed group of genes. In addition to MADS-box genes, several other co-expressed transcription factor genes were identified including those encoding Squamosa Binding Protein Like homolog 3, two zinc finger proteins and an MYB domain factor similar to *Antirrhinum MYB305*, an early regulator of the phenylpropanoid pathway.

*GRCD1*, which is already strongly upregulated in ray flowers at stage 3, continuing to stage 6, is co-expressed with the *TM3*-like MADS-box gene *G0000100021A03 *in all nine conditions (Figure 4b, Table 5). Two *SEPALLATA*-like genes also showed a highly correlated expression pattern. Furthermore, we identified a co-expressed gene encoding a MYB domain transcription factor similar to *Pisum *MYB26 [57] a pollen specific LIM domain and a NAM-like protein.

## Discussion

Nearly nothing is known about genetic mechanisms involved in flower-type specification, neither in Asteraceae nor in any other plant bearing heteromorphic flowers within a single genotype. We have defined the early stages of ray and disc flower development in *Gerbera hybrida *and have shown that although flower development initiates similarly in both flower types, differences in petal and stamen development can be identified at relatively young stages of inflorescence development. The large size of gerbera inflorescences as well as the presence of hundreds of coordinately developing flowers permitted us to microdissect individual flower primordia for RNA isolation and to compare gene expression during early stages of development. In general, we did not identify genes constitutively expressed throughout the chosen developmental stages, indicating that rapid transcriptional changes occur during very early stages of gerbera ray and disc flower development. The number of differentially expressed genes in our microarray comparisons increased dramatically with developmental time, which correlates with morphological differentiation as shown with SEM and histological analyses.

In our experiments we focused particularly on transcription factors that were differentially expressed in ray and disc flower primordia. Typical for disc flowers was the high number of genes putatively involved in mRNA processing and transcriptional regulation. We also identified several Squamosa Promoter Binding Protein homologs, the functions of which are still largely unknown even in *Arabidopsis*. HD-ZIP III type transcription factor genes similar to *REVOLUTA *were also upregulated in disc flowers. There are five genes encoding HD-ZIP III factors in *Arabidopsis*, *REVOLUTA, PHAVOLUTA, PHABULOSA, CORONA*, and *ATHB8*. During embryogenesis, *Arabidopsis *HD-ZIP III triple mutants fail to distinguish the central domain from the peripheral domain of the developing embryo, which leads to formation of a single, radially symmetric cotyledon [35]. Given this potential role in normally occurring asymmetry, it is tempting to speculate that the identified gerbera HD-ZIP III factors could function in regulation of symmetry at the inflorescence level. In ray flowers the most prominent group of upregulated transcription factor genes encode MADS-box regulatory proteins.

### Differences in stamen and petal development

Clear morphological differences between the flower types in stamen and petal development (Figures 2B and 2C) that begin at stage 5 probably reflect differences in cell division and elongation as well as in organ fusion. The most prominent difference is the arrest of stamen development in ray flowers. As also observed in gerbera, a common mechanism for generating unisexual flowers is the selective developmental arrest of either female or male organ primordia, which may occur at different stages of development in different species [58]. The most fundamental genes discriminating the sexes in flowering plants are the homeotic B- and C-class genes, which distinguish between carpel and stamen development [58]. Based on the ABC model of flower development, stamen identity is determined by activity of both B- and C-class MADS-box genes, while the activity of C-class genes alone controls carpel development [59]. However, several studies in eudicot species have failed to provide unambiguous support for a hypothesis that development of unisexual flowers would involve alterations in the expression patterns of the B- or C-function genes, which indicates that the genetic mechanisms in sex determination must act downstream of organ identity [60-64]. In gerbera as well, alterations in B- and C-class MADS-box gene expression are not likely the cause of arrested stamen development in ray flowers. Our results suggest that the role of the *SEP*-like gene *GRCD1*, highly upregulated in ray flowers, could be related to the flower-type specific arrest of stamen development. In ray flowers of transgenic plants in which *GRCD1 *expression was downregulated, sterile staminodes were converted into anthocyanin pigmented petals. However, stamen development was only slightly affected in bisexual disc flowers, where fertile pollen was produced although not released [1]. Altogether, these results suggest that the arrest of stamen development is connected to organ identity that is differentially established in ray versus disc flowers. We propose that whorl 3 organ identity establishment involves GRCD1 function, but only in ray flowers.

### Differential expression of genes encoding MADS domain transcription factors – flower-type specific regulatory complexes

**Figure 5:**
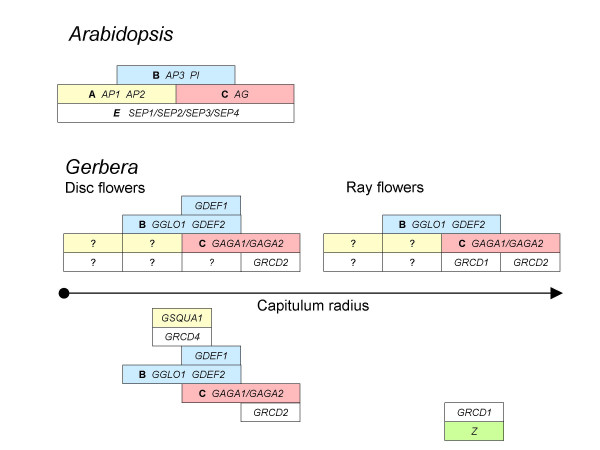
**A flower-type specific ABC(D)E model in *Gerbera***. In *Arabidopsis*, the genes *AP1*, *AP2*, *AP3*, *PI *and *AG *encode homeotic functions that are needed to determine identity of floral organs according to the ABC model [59]n addition to these genes, development of *Arabidopsis *floral organs requires the activity of a fourth function, labeled E in the extended 'quartet' model of flower development [74], which redundantly encoded by four related MADS box genes, *SEP1*, *SEP2*, *SEP3 *and *SEP4*. In *Gerbera*, the genes *GGLO1 *and *GDEF2 *are required for the B function. The C function is redundantly encoded by two highly similar genes, *GAGA1 *and *GAGA2*, but for the E function, we have observed subfunctionalization among *SEP*-like gene paralogs. In *Gerbera*, *GRCD1 *is needed for correct flower organ identity in whorl 3 of marginal female flowers only. Correspondingly, *GRCD2 *is necessary only for carpel development, with stronger loss-of-function phenotypes observed in the central disk flowers. Based on these observations, we predict that still unidentified (possibly redundant) E function MADS box genes are required in whorls 1 and 2, and in disc flower whorl 3. Furthermore, we have not yet identified *Gerbera *genes that might code for an A function active in whorls 1 and 2. These whorls react differentially to ectopic expression of *GAGA1 *or *GAGA2*, raising the possiblity that the A function may itself be split in *Gerbera*. Relative MADS box gene upregulation phenomena observed in our microarray experiments, organized along the capitulum radius (arrow), are summarized below (and formatted according to) the gerbera disc and ray flower ABCE models above. *Z *stands for the *TM3*-like gene *G0000100021A03*. GSQUA1 and GRCD1 expression in disc flowers is shown offset between whorls 2 and 3 to show their uncertain whorl-specificity regarding function.

The microarray data, as supported by real time RT-PCR, suggest that MADS-box proteins may be involved in specific complexes required for the differentiation of individual flower types during inflorescence development in Asteraceae. Differential gene expression of several gerbera MADS-box genes (*GGLO1*, *GDEF2, GAGA1*, *GAGA2 *and *GRCD1*) had earlier been detected with northern blots representing the same floral developmental stages used in this study (M. Kotilainen, unpublished data). The quantitative differences we observed at the gene expression level may reflect later qualitative protein-protein interaction differences and thereby the composition of specific protein complexes involved in regulation of organ identity in different flower types. Because our expression data revealed genes whose expression is correlated both spatially and temporally in various gerbera tissues, we are able to hypothesize specific protein-protein interactions, some of which have already been confirmed by yeast two-hybrid assays or are supported by transgenic phenotypes. A comprehensive interaction analysis of *Arabidopsis *MADS-box factors has shown that almost 100% of the interacting proteins have an overlap in expression pattern [56]. Coexpression can be taken as an indication for possible *in planta *interaction [56]. Furthermore, we have identified differential expression of new *SEP*-like genes, which in gerbera and other plants show a diversity of functions beyond those described for *Arabidopsis *[1,49,65]. Figure 5 summarizes our current view of gerbera organ-identity genes, indicating (above the arrow) specific factors that have shown flower-type specific effects in transgenic plants [46].

Upregulation of B- and C-class MADS-box genes as well as the *SEP*-like gene *GRCD2 *in central disc flowers probably reflects the major role these genes play in determining normal stamen and carpel identity (carpels only in case of *GRCD2*). In transgenic plants, suppression of B (GGLO1, GDEF2), C (GAGA1, GAGA2) or GRCD2 function caused similar phenotypes in both flower types [47,49]. Interestingly, the expression of *GSQUA1 *and the *SEP*-like gene *GRCD4 *correlated with B- and C-class gene expression. Despite its sequence similarity to Arabidopsis *APETALA1*, *GSQUA1 *is probably not involved in establishing the A function since its expression was detected at the base of developing flowers, in petals, and in developing vascular bundles of the capitulum [47,45]. However, *GRCD4 *was also upregulated during late petal development in our previous microarray studies [22], suggesting that it, together with B and C function genes, may encode a petal-specific *SEP *gene in gerbera. The function of *GDEF1*, a TM6-like B-class gene upregulated in disc flowers, is less clear. Unlike *GDEF2*, which is strongly expressed in both petals and stamens, *GDEF1 *is most highly expressed in stamens (S. Broholm, unpublished results) indicating function in stamen development. A highly similar gene in petunia (*PhTM6*) also showed stamen expression and strong protein-protein interaction with the petunia PHGLO2 polypeptide [66]. In gerbera, yeast two-hybrid results show that both GDEF1 and GDEF2 form heterodimers with the B function protein GGLO1 (data not shown), whereas neither protein interacts with GRCD2, the function of which is carpel-specific [49]. *GRCD1 *and the *TM3*-like gene *G0000100021A03 *are both highly upregulated in ray flowers. This suggests that these two genes may encode interacting proteins involved in the arrest of stamen development. Moreover, the expression of *GRCD1 *and *G0000100021A03 *correlated at the flower organ level across all nine conditions analyzed, suggesting that they may have additional functional roles, e.g., during petal development. Specific upregulation phenomena in disc versus ray flowers are summarized in Figure 5 below the arrow, the direction of which follows the capitulum radius.

### MADS protein complexes and a radial morphogenetic gradient

The capitulum of the Asteraceae has been used historically as a model to study inflorescence meristem development. Inflorescence determination and phyllotaxis have been investigated through wounding experiments, and flower-type specific organ development has been analyzed genetically. Both lines of research have suggested the action of a radial morphogenetic gradient in capitulum development. Cylindrical wounding of early sunflower inflorescences to produce isolated plugs of capitulum receptacle tissue resulted in the development of complete, miniature capitula embedded within larger capitula [67,68]. Thus, the radially organized sunflower receptacle can be reset into further radially patterned zones via disruption of cell-cell communication. Genetic studies of reproductive characters in *Microseris *also suggest cell-cell communication radially. The hairy and yellow achene (fruit) traits both show concentric localization in *Microseris *capitula, although they are independently regulated [69]. In the strains examined, hairy achenes (controlled by at least two genes) are always peripheral to smooth achenes, and there is a region of overlap ("half-hairy" achenes) in which individual cells appear to respond specifically to a defining gradient. The yellow-achenes trait also shows radial zonation, the extent of which differs among *Microseris *strains segregating for two yellow-achenes alleles. Bachmann and colleagues [70,69] have hypothesized that the genes responsible for peripheral hairy achenes are participants in the establishment of a radial morphogenetic gradient, and that the alleles of yellow-achenes have different response thresholds for this gradient.

Morphogenetic gradients with distinct patterning effects, such as production of ray and trans versus disc flowers in gerbera, can be set up by simple threshold models of short range activation and long range inhibition, so long as the feedback loops are non-linear [71]. An example of a non-linear relationship is an inhibited activator protein that must function as a dimer. MADS domain proteins form specific homo- and heterodimers and even higher-order complexes to conduct their function [53,54]. The long range (non-cell-autonomous) inhibitor could be a diffusible small molecular weight compound, but it has also been demonstrated that plant transcription factor proteins, including the *Antirrhinum *MADS domain factors DEF and GLO themselves, can move through plasmodesmata and act non-cell-autonomously [72,73]. Production of MADS multimers would add significantly to the possible responses to a morphogenetic gradient. In summary, the MADS protein system can provide a simple numerical scaffold (dimers, multimers) upon which to generate great cell, tissue, and organ diversity through differential regulation along a radius. This is well presented in development of the whorled structure of single floral meristems, but by extension, the same general radial gradient mechanisms might operate at the inflorescence level, as suggested by previous work on sunflower and *Microseris*. Since we have discovered that different MADS domain proteins are differentially expressed in ray versus disc flowers of gerbera (Fig. 5), we hypothesize that developmental control of the Asteraceae capitulum may be more than a simple analog of the single flower that it resembles. Comparative flower/inflorescence research on MADS-box gene transcriptional responses to candidate gradient-establishing molecules, as well as studies of *in vivo *protein-protein interactions, could help test this hypothesis. If corroborated, it will remain to be demonstrated whether gerbera capitula may have acquired flower-like developmental regulation secondarily, or whether their condensed structure makes a more general pattern easier to detect.

## Conclusion

Very little is known about the genetic mechanisms involved in flower type differentiation in Asteraceae. We have taken advantage of the large size of gerbera inflorescences to morphologicaly characterize developing ray and disc flower primordia. Although the development of individual flower types initiates similarly, differences in petal and stamen development are observed at relatively early stages. Global gene expression analyses using the gerbera cDNA microarray indicate that rapid transcriptional changes correlate with morphological differentiation of dividual flower types. Most interestingly, we identified several genes encoding MADS domain transcription factors that are differentially expressed in developing ray and disc flower primordia. We hypothesize that the quantitative and qualitative expression differences discovered reflect control by a radial morphogenetic gradient across the capitulum that may lead to formation of specific MADS protein complexes that regulate the differentiation of individual flower types. Based on coexpression, we propose functional hypotheses for several MADS-box transcription factors that will be further tested in protein-protein interaction studies and using transgenic gerbera plants.

## Methods

### Scanning Electron Microscopy (SEM)

Young inflorescences were fixed, dried in a critical point drying unit and coated with platinum/palladium [49]. Samples were examined through a scanning electron microscope (Zeiss DSM 962) in the Electron Microscopy Laboratory of the Institute of Biotechnology at the University of Helsinki.

### Light microscopy

Young inflorescences were fixed in FAA (50% ethanol, 5% acetic acid, 2% formaldehyde) and transferred through ethanol series into 100% xylene. Samples were embedded in paraffin and cut into 10 μm sections. For histological staining, paraffin was removed with xylene and the sections were stained with saffranin (1% in water) and aniline blue (1% in 50% ethanol). The sections were observed and photographed under a light microscope (Olympus CX41).

### Sample collection and RNA isolation

Flower primordia of ray and disc flowers were isolated with a scalpel under stereomicroscope and immediately stored in liquid nitrogen. RNA was isolated using Trizol reagent (Invitrogen, Carlsbad CA, USA) with scaled down protocol. At least 10 inflorescences, each bearing hundreds of primordia, were collected and pooled together for RNA isolation in each stage. Each pool included more than one thousand individual flower primordia. Pooling of the samples was used in order to minimize biological variation between samples instead of real biological replicates which were not an option due to the limited amount of material. Each experiment included at least two RNA isolations that were pooled together. Quality and the yield of RNA was analyzed with Agilent 2100 Bioanalyzer (Agilent Technologies, Waldbronn, Germany) using RNA 6000 Nano LabChip^® ^kit (Agilent Technologies, Waldbronn, Germany) and RNA 6000 ladder (Ambion, Austin, TX, USA).

### Microarray experiments

Microarray experiments followed MIAME standards and can be viewed at the ArrayExpress database. Amplification and labelling of the samples were done using Amino Allyl Message Amp Kit (Ambion, Austin, TX, USA) starting with 2 μg of total RNA. In this total RNA sample several RNA isolations had been pooled together, as described above. Total RNA was isolated from the pools of flower primordia. When more than one amplification was required to get enough amplified RNA (aRNA), several amplifications were done and pooled together. For one hybridization, 7.5 μg of aRNA was further labelled with Cy3 or Cy5 fluorescent dyes (Amersham Biosciences, Little Chalfont Buckinghamshire, UK). For each developmental stage, 4 technical replicates were included from which two were dye-swapped. In practice, several hybridizations were done per each stage and the four technical replicates selected for the analysis were chosen to be of high quality. Prehybridization, hybridization and washes followed previous protocols [22]. After hybridization, slides for stages 3 and 5 were scanned with GSI Lumonics ScanArray 5000 (Packard/Perkin Elmer) and for stage 6 using Genetix 5000 AL scanner (Genetix, New Milton, Hampshire, UK) to produce two gray-scale TIFF images. In the GSI Lumonics ScanArray, laser power was changed in order to adjust the two channels whereas in the Genetix 5000, laser value was kept at 100% and PMT gain was changed in order to adjust the two channels. The resolution was 10 μm in all cases.

### Microarray data analysis

Genepix pro 5.0 (Axon Instruments, USA) was used to localize the spots and quantify the intensity values in all experiments. The local feature background median was used to subtract the background noise, and visually flawed spots were filtered out from further analysis. Data were normalized and filtered using GeneSpring 7.2 (Silicon Genetics). Due to the non-linearity of the data Lowess normalization was applied. Features with a higher background signal than expression signal were excluded from further analysis. After this the following statistical analysis was performed to find transcripts with significantly altered expression levels between the two samples across four replicates. The data was filtered using one-sample t-test. In order to find genes that were differentially expressed, a t-test was performed for each developmental stage (3, 5 and 6) independently. P-values were adjusted for multiple comparisons by the Benjamini and Hochberg false discovery rate method that controls the Type I family-wise error, and genes having corrected p-value < 0.05 were selected as statistically significant. Here, using one-sample t-test, one can select genes that were differentially expressed between disc and ray flowers, because RNA extracted from both flower types were hybridized on the same chip. Therefore, when the t-test p-value was non-significant, the gene was not differentially expressed, and it was excluded from further analysis. Furthermore, in addition to the p-value criterion a transcript had to show more than 1.2-fold change in order to be considered as differentially expressed.

### Data mining of co-expressed transcripts with GAGA1 and GRCD2

Three conditions, stage 3, 5 and 6, from the experiments reported here were merged with 6 previously done microarray comparisons [22] to form a combined data set with nine conditions. The previously performed 6 conditions include gene expression analysis of various flower organs (early and late petal development, pappus bristles, stamen and flower scape) that were compared against a pooled reference sample. Comparison of inflorescence sample to leaf sample was also included. These experiments have been described in [22] and the data is available at the ArrayExpress database with accessions E-MEXP-206 and E-MEXP-207. After the data from all nine conditions were merged, the experiment was normalized in GeneSpring 7.2 (Silicon Genetics) using Lowess normalization. Similarity in expression patterns across the various conditions was used as the criterion for co-expression and was calculated using standard correlation in GeneSpring. Mathematically, standard correlation is very similar to Pearson correlation, but it measures the angular separation of expression vectors around zero instead of around one. The transcripts that were functionally annotated to the class 'transcription' were included in the analysis. Transcripts that had a correlation coefficient of more than 0.80 with microarray probes GAGA1 (most highly upregulated in disc flowers) or with GRCD1 (most highly upregulated in ray flowers) were considered to be similarly expressed.

### Real time RT-PCR

Altogether, 20 transcripts that were printed on the gerbera cDNA microarray were further verified using real time RT-PCR. Total RNA (1.5 μg) was used for cDNA synthesis using TaqMan reverse transcription kit (Applied Biosystems, Roche Molecular Systems Inc., New Jersey, USA) and 5 μl of cDNA was used as a template in PCR reactions. Primers were designed using PrimerExpress software (Applied Biosystems, Foster City, CA, USA). PCR was done in triplicate with 50 nM of primers using ABI 7700 sequence detection systems (Applied Biosystems, Foster City, CA, USA) cycling conditions as default. Ubiquitin was used as a standard against which the raw threshold values (Ct) were normalized in order to get ?Ct values (normalized Ct values). Expression ratio was calculated using the real expression values which in turn were calculated from the formula 2^**ΔCt**^. Each plate included separate ubiquitin standards.

### Accession numbers

Microarray array design used in this article can be found at the Array-Express arrayexpress under accession number A-MEXP-244 and A-MEXP-249 and experiment data under accession E-MEXP-418. Previous microarray results [22], included in the analysis presented in this paper, can be viewed under accessions E-MEXP-206 and E-MEXP-207. EST sequence data from this article can be found in the EMBL/GenBank data libraries under accession numbers AJ750001-AJ766994. EST sequence data and annotations are also available in OpenSputnik database at  and at 

## Authors' contributions

RAEL and PE conceived and designed the experiments. RAEL and SB carried out the experiments. RAEL analyzed the data. THT and PE contributed reagents. RAEL, VAA and PE drafted the manuscript. All authors read and approved the final manuscript.

## Supplementary Material

Additional File 1 **Table A1**. Verification of the microarray results with real time RT-PCR analysis done for 12 randomly selected transcripts. Click here for file

Additional File 2 **Table A2**. Verification of the microarray results with real time RT-PCR analysis done for 8 MADS-box genes in different developmental stages of disc and ray flower primordia. Click here for file

## Figures and Tables

**Table 1:** Genes that are significantly more expressed in disc or in ray flowers at developmental stage 3 with p-value >0.05 and fold change >1.2.

**Probe name**	**ratio**	**p-value**	**Description**
**Disc flower stage 3**			

G0000200053G12	1.84	0.0211	Gene for 26S ribosomal RNA
G0000800014B4	1.4	0.0211	Novel Unknown protein 3123
G0000100036F9	6.0	0.0268	Vegetative storage protein, VSP
G0000100002C3	1.5	0.0309	Novel Unknown protein 0028
G0000700006H7	2.9	0.0322	Major latex-like protein
G0000200017H2	2.3	0.0322	Putative cytochrome P-450
G0000200019E3	4.0	0.0324	Omega-6 fatty acid desaturase
G0000200014G1	1.3	0.0324	Glucose acyltransferase
G0000600014B12	1.3	0.0358	Acid phosphatase
G0000200056F6	1.4	0.0425	Putative ADP-ribosylation factor
G0000200048F4	1.5	0.0425	Polyubiquitin
G0000900011F6	1.6	0.0425	Novel Unknown protein 4198
G0000200033D2	1.8	0.0425	Unknown protein 0409
G0000600002F9	1.5	0.0425	Clathrin assembly protein
G0000500007D8	2.4	0.0489	Dehydration-responsive protein RD22

**Ray flower stage 3**			

G0000800020B7	1.7	0.0211	Unknown protein
G0000400024B9	1.7	0.0268	Tubulin beta-7 chain
G0000200033F6	1.4	0.0268	Novel Unknown protein 1096
G0000100025H5	2.0	0.0268	SOUL-like protein
G0000200018D11	3.3	0.0268	Flower-specific gamma-thionin precursor
G0000200049B9	1.4	0.0322	Novel Unknown protein 1280
G0000200005C1	2.5	0.0322	Unknown protein 0241
G0000200019D8	2.0	0.0358	Novel Unknown protein 0858
G0000300010D2	1.7	0.0388	Novel Unknown protein 1660
G0000200009B2	2.0	0.0425	Novel Unknown protein 0638
PGTY3 XHOG1	2.5	0.0425	*G. hybrida *MADS-box protein, GRCD1
G0000700003H5	2.2	0.0489	Unknown protein

**Table 2:** Genes that are significantly more expressed in disc or in ray flowers in both stages 5 and 6 with p-value >0.05 and ratio>1.2.

**Probe name**	**Fold change**			**Description**	**Functional group**
	**st5**	**st6**	**p-value**		

**Common to disc flower stages 5 and 6**					

G0000700005H5	3.6	2.2	0.0330	Putative GDSL-motif lipase	Metabolism
G0000200013C10	2.1	1.9	0.0404	*G. hybrida *MADS-box protein, GAGA1	Transcription
G0000200001H1	1.5	1.7	0.0423	Nucleoid DNA-binding-like protein	
G0000400020F7	1.3	1.3	0.0427	Putative cysteine protease	Protein destination and storage
G0000600005D3	2.4	1.5	0.0423	Ubiquitin-conjugating enzyme E2-17 kDa 9 (EC 6.3.2.19)	
G0000400019A2	2.8	1.6	0.0400	Putative fibrillin	Transport
G0000700012A7	3.0	2.1	0.0369	Alpha-expansin 1	Cell structure
G0000200003F1	1.6	1.7	0.0330	13-lipoxygenase (EC 1.13.11.12)	
G0000200002B2	1.6	2.6	0.0444	Putative senescence associated protein	Signal transduction
G0000200002H10	2.3	2.1	0.0330	RAC GTP-binding protein ARAC7	
G0000200004B5	2.0	2.4	0.0391	Putative beta-1,3-glucanase	Disease and defence
G0000700014A3	2.0	1.3	0.0391	Wound-inducible carboxypeptidase (EC 3.4.16.5)	
G0000200027D4	2.1	1.2	0.0489	Putative S-adenosyl-methionine-sterol-c-methyltransferase	
G0000800013E11	2.5	2.1	0.0458	*G. hybrida *chalcone synthase, GCHS1	Secondary metabolism
G0000100014H8	3.2	1.6	0.0391	Flavonol synthase (EC 1.14.11.-)	
G0000600015H10	2.0	2.3	0.0330	Lipid-transfer protein	LTP
G0000600006A10	6.9	7.8	0.0339	Lipid-transfer protein	
G0000300001A5	15.7	8.1	0.0330	Lipid-transfer protein	
G0000500014H11	2.5	1.5	0.0362	Nonspecific lipid-transfer protein precursor	
PHTT660F6	2.1	1.7	0.0330	*G. hybrida *ubiquitin-like protein GUB1	

**Common to ray flower stages 5 and 6**					

G0000600019C3	3.3	3.3	0.0369	Beta-d-glucosidase precursor (EC 3.2.1.21)	Metabolism
G0000700006B12	1.4	1.7	0.0446	CER1 protein (aldehyde decarbonylase)	
G0000800008A7	1.7	1.3	0.0455	ATP citrate lyase b-subunit (EC 4.1.3.8)	Energy
G0000500009D8	1.7	1.7	0.0416	Putative SB21 mRNA fibrillarin protein	Transcription
G0000600018E9	1.4	1.4	0.0423	40S ribosomal protein S11	Protein synthesis
G0000400012D8	1.7	1.4	0.0437	60s ribosomal protein L34	
G0000400007C8	1.4	1.7	0.0369	60S ribosomal protein L26	
G0000500018B6	1.7	1.4	0.0490	Quercus suber 60S ribosomal protein L41	
G0000600017A7	2.0	1.7	0.0419	60S ribosomal protein L38	
G0000500005H6	2.0	0.7	0.0330	Elongation factor 2	
G0000900002H11	1.4	1.1	0.0369	Similar to TCP1-chaperonin cofactor A	Protein destination and storage
G0000400003B11	1.7	1.7	0.0369	Ubiquitin extension protein	
G0000600014B5	2.5	2.5	0.0351	GASA-like protein mRNA	Cell structure
G0000600020B2	2.0	1.7	0.0459	Homologous early light induced protein	Disease and defence
G0000200014F9	2.0	1.7	0.0314	Remorin	
G0000800005B5	1.4	1.3	0.0314	Cofactor-independent phosphoglyceromutase (EC 5.4.2.1)	
G0000700014E10	3.3	1.4	0.0330	Polyphenol oxidase precursor (EC 1.10.3.1)	Secondary metabolism
G0000800018F9	2.0	1.4	0.0369	Dalcochinin beta-glucosidase (EC 3.2.1.21)	
G0000600018F10	2.5	2.0	0.0211	Nonspecific lipid-transfer protein (LTP)	LTP

**Table 3:** Genes annotated to the class of "transcription" that show differential expression in disc flower at stages 5 and 6 in comparison to ray flowers, with p-value >0.05 and fold change >1.2.

**Probe name**	**fold change**	**p-value**	**Description**
**Disc flower stage 5**			

G0000500014H10	1.9	0.0369	Putative DNA-binding protein, bHLH transcription factor
G0000100025H8	3.4	0.0391	Putative RING zinc-finger protein
G0000200013C10	2.1	0.0404	*G. hybrida *MADS-box protein, GAGA1
G0000200001H1	1.5	0.0423	Nucleoid DNA-binding-like protein
G0000600012E9	2.2	0.0423	mRNA CAP methyltransferase-like protein

**Disc flower stage 6**			

G0000200005F5	3.2	0.0075	Putative ATP-dependent RNA helicase A
G0000200001H1	1.7	0.00773	Nucleoid DNA-binding-like protein
G0000400013B5	1.3	0.00857	Homeobox protein HD-ZIP
G0000100023F8	1.8	0.00857	Putative spliceosome-associated protein
G0000200006H5	1.9	0.00875	Putative TATA binding protein-associated factor
G0000200029A8	2.0	0.00959	Putative splicing factor
G0000200047A7	1.6	0.0106	Squamosa Promoter Binding Protein-homologue 3
G0000800003E2	1.9	0.0139	YABBY 3
G0000100028F12	1.8	0.0139	RNA helicase
PDY55-10E1	1.7	0.0139	*G. hybrida *MADS-box protein, GAGA2
G0000200054C5	1.4	0.0140	DR1-like protein
G0000700008F1	1.5	0.0141	YABBY 3
G0000200016E6	1.3	0.0148	Zinc-finger protein-like
G0000200009B7	1.4	0.0151	Homeobox protein HD-ZIP
G0000200004G9	3.1	0.0155	Squamosa Promoter Binding Protein-like 4
G0000200013C10	1.9	0.0173	*G. hybrida *MADS-box protein, GAGA1
G0000100027C12	1.3	0.0186	Transcription initiation factor IIA gamma chain
PDY55-2D1	1.8	0.0209	*G. hybrida *MADS-box protein, GAGA1
PDY8-4H1	1.7	0.0214	*G. hybrida *MADS-box protein, GRCD2
G0000800002C9	1.3	0.0223	*G. hybrida *MADS-box protein
G0000400007A1	1.3	0.0224	Transcriptional activator DEMETER
G0000400023B9	1.5	0.0227	Putative WRKY transcription factor 30
G0000700003D2	1.3	0.0231	Poly(A)-binding protein
G0000100034F2	1.3	0.0242	bHLH transcription factor gbof-1
G0000200004G12	1.8	0.0265	Putative RNA-binding protein
G0000700016H8	1.7	0.0300	mRNA CAP methyltransferase-like protein
G0000700015A6	1.4	0.0323	RNA-binding protein MEI2, putative
PHEP7F1	1.3	0.0334	*G. hybrida *MADS-box protein, GSQUA1
G0000400005E2	1.3	0.0374	WRKY transcription factor NT-SUBD48
G0000400006C3	1.3	0.0405	Glycine-rich RNA binding protein 2 (GRP2)
G0000900002D1	1.8	0.0409	*G. hybrida *MADS-box protein, GDEF1

**Table 4:** Genes annotated to the class of "transcription" that show differential expression in ray flowers at stages 5 and 6 in comparison to disc flowers, with p-value >0.05 and fold change >1.2.

**Probe name**	**fold change**	**p-value**	**Description**
**Ray flower stage 5**			

G0000600015D9	2.0	0.0404	Chromatin remodelling complex ATPase chain ISWI protein
G0000800020B7	1.7	0.0405	Putative RNA-binding protein
G0000500009D8	1.7	0.0416	Putative SB21 mRNA fibrillarin protein
G0000600012F9	1.7	0.0427	Metallothionein-I gene transcription activator
G0000600010E3	2.0	0.0432	Metallothionein-I gene transcription activator
G0000300020A5	1.4	0.0432	Post-transcriptional gene silencing protein, Argonaute-like
G0000800017E8	1.7	0.0446	Transcription regulator

**Ray flower stage 6**			

G0000300001F12	1.7	0.00857	Putative transcription factor BTF3
G0000700003A3	1.4	0.00891	*G. hybrida *MADS-box protein, GRCD5
G0000200011H1	1.4	0.0110	Putative DNA-directed RNA polymerase subunit
G0000500018F11	1.4	0.0112	RNA-binding protein
G0000700009F4	1.7	0.0129	Nucleoid DNA-binding-like protein
G0000200014A7	1.4	0.0140	*G. hybrida *MADS-box protein, GRCD3
G0000500013C9	1.4	0.0141	Putative transcription factor BTF3
G0000400010C8	1.4	0.0144	Putative CCR4-associated factor protein
G0000200011F5	1.7	0.0156	Nucleoid DNA-binding-like protein
G0000200055E9	1.7	0.0157	Putative SB21 mRNA fibrillarin protein
G0000500013B9	1.7	0.0160	GATA-1 zinc-finger protein
G0000500009D8	1.7	0.0170	Putative SB21 mRNA fibrillarin protein
G0000100020B8	1.4	0.0172	Putative C2H2-type zinc-finger protein
PGTY3 XHOG1	2.5	0.0174	*G. hybrida *MADS-box protein, GRCD1
G0000700013E1	1.4	0.0184	DNA-binding protein ACBF
G0000500019B2	1.7	0.0215	Putative dimethyladenosine transferase
G0000600012D7	2.0	0.0227	*G. hybrida *bHLH transcription factor, GMYC1
G1-2C11D2	2.0	0.0229	*G. hybrida *MADS-box protein, Tm3-like
G0000100001D9	1.4	0.0242	DNA-binding protein GBP16
G0000600011G6	1.4	0.0245	Zinc-finger protein CONSTANS-like 3
G0000600004G6	1.4	0.0267	Poly(A)-binding protein
G0000100015G4	2.5	0.0271	*G. hybrida *MADS-box protein, GRCD1
G0000200035H11	1.4	0.0274	NUM1 protein
G0000500016H3	1.4	0.0303	Putative transcription factor BTF3
G0000400014B2	2.0	0.0331	LIM-domain protein PLIM-2
G0000200045F4	1.4	0.0358	Transducin/WD-40 repeat protein-like
G2-14A07E2	1.4	0.0364	*G. hybrida *MADS-box protein, GRCD3
G0000200001C6	1.4	0.0365	MADS-box transcription factor CDM51
G1-21A03C2	1.4	0.0366	*G. hybrida *MADS-box protein
G0000400013D12	1.4	0.0387	Putative CCAAT-box-binding trancription factor
G0000200049G11	1.4	0.0395	Heat shock transcription factor 31

**Table 5:** Genes co-expressed with the MADS domain factors *GAGA1 *(upregulated in disc flowers) and *GRCD1 *(upregulated in ray flowers) across 9 different microarray experiments when correlation coeffiecient >0.8 was used as criterion.

**Correlation with GAGA1**		
**Probe name**	**Correlation**	**Annotation**

PDY55-2	1	*G. hybrida *MADS-box protein, GAGA1
PDY8-4	0.963	*G. hybrida *MADS-box protein, GRCD2
G0000700006A2	0.943	*G. hybrida *MADS-box protein, GDEF2-like
PDY119-4	0.936	*G. hybrida *MADS-box protein, GDEF2
G0000100028F12	0.935	RNA helicase
PDY55-10	0.922	*G. hybrida *MADS-box protein, GAGA2
PDY55-8	0.921	*G. hybrida *MADS-box protein, GDEF1
G0000200013C10	0.917	*G. hybrida *MADS-box protein, GAGA1
G0000100027H2	0.913	MADS-box protein, GGLO1
G0000200001H1	0.903	Nucleoid DNA-binding-like protein.
G0000400013D7	0.892	*G. hybrida *MADS-box protein, GDEF2
G0000200011D6	0.887	*G. hybrida *MADS-box protein, GGLO1
G0000100019F6	0.884	Putative VIP1 protein
G1-5B	0.882	*G. hybrida *MADS-box protein, GRCD4
PDY55-9	0.876	*G. hybrida *MADS-box protein, GGLO1
G0000200002A7	0.856	CHP-rich zinc-finger protein-like
G0000700002E7	0.850	Squamosa Promoter Binding Protein-homologue 3
G0000100021C10	0.840	Putative transcription factor
G0000200059G3	0.838	Putative PRP19-like spliceosomal protein
G0000200004G12	0.834	Putative RNA-binding protein
G0000100022G6	0.832	MYB-related protein 305
G0000400001G12	0.831	MADS-box protein, GRCD2
G0000200006H5	0.829	Putative TATA binding protein-associated factor
G0000300021A5	0.818	Putative RING zinc-finger protein
PHEP7	0.805	*G. hybrida *MADS-box protein, GSQUA1

**Correlation with GRCD1**		

**Probe name**	**Correlation**	**Annotation**

PGTY3 XHO	1	*G. hybrida *MADS-box protein, GRCD1
G0000100015G4	0.988	MADS-box protein, GRCD1
G0000300001D6	0.929	MYB26
G0000700011D8	0.915	Pollen specific LIM-domain protein 1B
G0000800020B7	0.902	Putative RNA binding protein
G0000500017B2	0.879	NAM-like protein
G0000200011F5	0.861	Nucleoid DNA-binding-like protein
G1-21A03	0.834	*G. hybrida *MADS-box protein, Tm3-like
G0000700003A3	0.826	MADS-box protein, GRCD5
G0000100001D9	0.814	DNA-binding protein, GBP16.
G0000200014A7	0.801	MADS-box protein, GRCD3
