# Supplementary material for: Patterns of MADS-box gene expression mark flower-type development in Gerbera hybrida (Asteraceae)
Source: BMC Plant Biol. 2006 Jun 9;6:11. doi: 10.1186/1471-2229-6-11 (PMC1525168; doi:10.1186/1471-2229-6-11)
Supplement: Additional File 1 — Table A1. Verification of the microarray results with real time RT-PCR analysis done for 12 randomly selected transcripts. [file 1471-2229-6-11-S1.doc]

|  |  | **∆Ct** | | | | **2^∆Ct^** | | | | **q (RT)-PCR DF/RF** | | **Microarray DF/RF** | |
| --- | --- | --- | --- | --- | --- | --- | --- | --- | --- | --- | --- | --- | --- |
| **Gene** | **annotation** | **RF st3** | **DF st3** | **RF st5** | **DF st5** | **RF st3** | **DF st3** | **RF st5** | **DF st5** | **St 3** | **St 5** | **St3** | **St 5** |
| **G6-4E8** | Initiation factor 5A (eIF-5A) | 8.3 | 6 | 7.9 | 7.5 | 315.2 | 64 | 238.9 | 181 | 4.9 | 1.3 | 1.7 | 1.2 |
| **G5-10H9** | heat shock protein 70 | 6.2 | 3 | 5.4 | 5 | 73.52 | 8 | 42.22 | 32 | 9.2 | 1.3 | 2.4 | 1.1 |
| **G8-5C3** | umor protein homolog (tctp) | -1.7 | -2.9 | -3.1 | -2.1 | 0.31 | 0.13 | 0.12 | 0.23 | 2.3 | 0.5 | 0.9 | 0.6 |
| **G6-15D9** | putative chromatin remodelling complex atpase chain iswi protein | 1.3 | 1.3 | 0.1 | 2.4 | 2.46 | 2.46 | 1.07 | 5.28 | 1 | 0.2 | 0.9 | 0.5 |
| **G6-19C3** | beta-d-glucosidase precursor | 1.5 | 1.5 | 0.2 | 2.2 | 2.83 | 2.83 | 1.15 | 4.60 | 1 | 0.3 | 0.6 | 0.3 |
| **G7-14E10** | polyphenol oxidase precursor | 2.8 | 3 | 4 | 4.9 | 6.96 | 8 | 16 | 29.86 | 0.9 | 0.5 | 0.2 | 0.9 |
| **G7-6H7** | major latex-like protein | 2.8 | 1.5 | 1.5 | 1.1 | 6.96 | 2.82 | 2.83 | 2.14 | 2.5 | 1.3 | 2.9 | 5.1 |
| **G2-18D11** | flower-specific gamma-thionin precursor (defensin sd2) | -0.3 | 0.3 |  |  | 0.81 | 1.23 |  |  | 0.7 |  | 0.3 |  |
| **G7-16E9** | similar to developmental protein dg1118 | 3.2 | 4.8 | 3.7 | 5.5 | 9.19 | 27.86 | 13 | 45.25 | 0.3 | 0.3 | 0.5 | 1.3 |
| **G7-11D8** | pollen specific lim domain protein 1b | 3.8 | 3.3 | 2.3 | 4.4 | 13.93 | 9.85 | 4.93 | 21.11 | 1.4 | 0.2 | 0.6 | 0.8 |
| **G2-44H10** | putative serine protease | 4.3 | 4.4 | 4.6 | 4.9 | 19.7 | 21.11 | 24.25 | 29.86 | 0.9 | 0.8 | 0.6 | 0.6 |
| **G2-29E1** | mads box transcription factor | 2 | 2.3 | 0.8 | 3.7 | 4 | 4.93 | 1.74 | 13 | 0.8 | 0.1 | 1 | 0.7 |

**Table A1.** Verification of the microarray results with real time RT-PCR analysis done for 12 randomly selected transcripts.

.
