# Supplementary material for: Patterns of MADS-box gene expression mark flower-type development in Gerbera hybrida (Asteraceae)
Source: BMC Plant Biol. 2006 Jun 9;6:11. doi: 10.1186/1471-2229-6-11 (PMC1525168; doi:10.1186/1471-2229-6-11)
Supplement: Additional File 2 — Table A2. Verification of the microarray results with real time RT-PCR analysis done for 8 MADS-box genes in different developmental stages of disc and ray flower primordia. [file 1471-2229-6-11-S2.doc]

|  |  | **∆ Ct** | | | **2^∆Ct^** | | **Q(RT)-PCR DF/RF** | **Micro**  **array DF/RF** |
| --- | --- | --- | --- | --- | --- | --- | --- | --- |
| **Gene** | **Annotation** | **RF st3** | **DF st3** | | **RF st3** | **DF st3** | **St 3** | **St 3** |
| gglo1 | Gerbera hybrida MADS-box protein GGLO1 | -0.4 | -0.1 | | 0.758 | 0.933 | 0.81 | 1.63 |
| gdef2 | Gerbera hybrida MADS-box protein GDEF2 | 7.4 | 7.8 | | 168.9 | 222.9 | 0.76 | 1.35 |
| grcd1 | Gerbera hybrida MADS-box protein GRCD1 | 3.89 | 5.6 | | 14.83 | 48.5 | 0.31 | 0.4 |
| gaga2 | Gerbera hybrida MADS-box protein GAGA2 | 12.49 | 12 | | 5753 | 4096 | 1.40 | 1.62 |
| gdef1 | Gerbera hybrida MADS-box protein GDEF1 | 5.59 | 5.7 | | 48.17 | 51.98 | 0.93 | 1.16 |
| grcd2 | Gerbera hybrida MADS-box protein GRCD2 | 3.29 | 3.6 | | 9.781 | 12.13 | 0.81 | 1.49 |
| gaga1 | Gerbera hybrida MADS-box protein GAGA1 | 10.49 | 10.3 | | 1438 | 1261 | 1.14 | 1.36 |
| g2-29e1 | Gerbera hybrida MADS-box protein | 1.79 | 2.7 | | 3.458 | 6.498 | 0.53 | 0.97 |
|  |  |  |  | |  |  |  |  |
|  |  |  |  | |  |  |  |  |
|  |  | **∆Ct** | | | **2^∆Ct^** | | **q(RT)-PCR DF/RF** | **Micro**  **array DF/RF** |
| **Gene** | **Annotation** | **RF st5** | | **DF st5** | **RF st5** | **DF st5** | **St 5** | **St 5** |
| gglo1 | Gerbera hybrida MADS-box protein GGLO1 | -0.3 | | -1.1 | 0.812 | 0.467 | 1.74 | 1.6 |
| gdef2 | Gerbera hybrida MADS-box protein GDEF2 | 8.4 | | 6.8 | 337.8 | 111.4 | 3.03 | 1.47 |
| grcd1 | Gerbera hybrida MADS-box protein GRCD1 | 4.4 | | 4.2 | 21.11 | 18.38 | 1.15 | 0.79 |
| gaga2 | Gerbera hybrida MADS-box protein GAGA2 | 12.8 | | 11.7 | 7132 | 3327 | 2.14 | 1.67 |
| gdef1 | Gerbera hybrida MADS-box protein GDEF1 | 6.9 | | 5.5 | 119.4 | 45.25 | 2.64 | 1.3 |
| grcd2 | Gerbera hybrida MADS-box protein GRCD2 | 4.4 | | 3.2 | 21.11 | 9.19 | 2.30 | 2 |
| gaga1 | Gerbera hybrida MADS-box protein GAGA1 | 11.9 | | 10.4 | 3822 | 1351 | 2.83 | 2 |
| g2-29e1 | Gerbera hybrida MADS-box protein | 2.4 | | 2.7 | 5.278 | 6.498 | 0.81 | 0.65 |

**Table A2.** Verification of the microarray results with real time RT-PCR analysis done for 8 MADS-box genes in different developmental stages of disc and ray flower primordia.
